# Prevention and treatment of neonatal nosocomial infections

**DOI:** 10.1186/s40748-017-0043-3

**Published:** 2017-02-13

**Authors:** Jayashree Ramasethu

**Affiliations:** Division of Neonatal Perinatal Medicine, Department of Pediatrics, MedStar Georgetown University Hospital, Washington DC, 20007 USA

**Keywords:** Nosocomial, Infection, Newborn, Prevention, CLABSI, VAP

## Abstract

Nosocomial or hospital acquired infections threaten the survival and neurodevelopmental outcomes of infants admitted to the neonatal intensive care unit, and increase cost of care. Premature infants are particularly vulnerable since they often undergo invasive procedures and are dependent on central catheters to deliver nutrition and on ventilators for respiratory support. Prevention of nosocomial infection is a critical patient safety imperative, and invariably requires a multidisciplinary approach. There are no short cuts. Hand hygiene before and after patient contact is the most important measure, and yet, compliance with this simple measure can be unsatisfactory. Alcohol based hand sanitizer is effective against many microorganisms and is efficient, compared to plain or antiseptic containing soaps. The use of maternal breast milk is another inexpensive and simple measure to reduce infection rates. Efforts to replicate the anti-infectious properties of maternal breast milk by the use of probiotics, prebiotics, and synbiotics have met with variable success, and there are ongoing trials of lactoferrin, an iron binding whey protein present in large quantities in colostrum. Attempts to boost the immunoglobulin levels of preterm infants with exogenous immunoglobulins have not been shown to reduce nosocomial infections significantly. Over the last decade, improvements in the incidence of catheter-related infections have been achieved, with meticulous attention to every detail from insertion to maintenance, with some centers reporting zero rates for such infections. Other nosocomial infections like ventilator acquired pneumonia and staphylococcus aureus infection remain problematic, and outbreaks with multidrug resistant organisms continue to have disastrous consequences. Management of infections is based on the profile of microorganisms in the neonatal unit and community and targeted therapy is required to control the disease without leading to the development of more resistant strains.

## Background

Advances in neonatal care have lead to the increasing survival of smaller and sicker infants, but nosocomial infections (NI), also known as health care associated or hospital acquired infections continue to be a serious problem. Late-onset sepsis (LOS), or sepsis acquired after 72 h of life, with the exception of Group B streptococcal or Herpes simplex virus infection, is usually hospital acquired, particularly in infants who are hospitalized from birth. These infections are associated with increased mortality rates, immediate and long term morbidity, prolonged hospital stay and increased cost of care [1–3]. Efforts to eradicate neonatal NI have had limited success in some areas, but many remain intransigent, and outbreaks with multi – drug resistant organisms (MDRO) continue to occur in neonatal intensive care units (NICUs) worldwide.

## Risk of NI in preterm, late preterm and term infants

Prematurity is the most important risk factor for NI. In the United States, surveillance data over almost 2 decades from the National Institute of Child Health and Human Development (NICHD) Neonatal Network show that 20–25% of very low birth weight (VLBW, birth weight ≤ 1500 g) infants who survived beyond 3 days were found to have one or more episodes of blood culture proven sepsis, with the majority being caused by gram-positive organisms, predominantly coagulase-negative staphylococci (CONS) (Table 1) [1–3]. The rate of infections was inversely related to birth weight and gestational age, with 50% of the infections occurring in infants born at <25 weeks or weighing less than 750 g at birth. Considerable center to center variability in the incidence of late-onset sepsis has been noted with rates of LOS ranging from 10.6 to 31.7%, despite adjusting for birth weight, GA, race and sex [2].

**Table 1:** Distribution of organisms responsible for late-onset sepsis

Organism	VLBW infants NICHD NRN 1991–1993^1^	VLBW infants NICHD NRN 1998–2000^2^	VLBW infants NICHD NRN 2002–2008^3^
Incidence of LOS	25	21	25
**Gram-positive**			
Staphylococcus coagulase-negative	55	48	53
Staphylococcus aureus	9	8	11
Enterococcus/Group D strep	5	3	4
Group B streptococcus	2	2	2
Other	2	9	7
**Gram-negative**			
Enterobacter	4	3	3
Escherichia coli	4	5	5
Klebsiella	4	4	4
Pseudomonas	2	3	2
Other	4	1	2
**Fungi**			
Candida albicans	5	6	5
Candida parapsilosis	2	4	2
Other	2	2	1

Numbers are expressed in percentages

*Abbreviations NICHD NRN* National Institutes of Child Health and Human Development Neonatal Research Network, *VLBW* Very low birth weight, birth weight ≤1500 g

There has been some progress recently in tackling neonatal NI. NICHD surveillance data showed that rates of LOS decreased from 2005 to 2012 for infants of each gestational age, (eg for infants born at 24 weeks, it decreased from 54 to 40%, and for those born at 28 weeks, the decrease was from 20 to 8%) [4]. Comparable decreases in the rates of LOS in preterm VLBW infants was noted in 669 North American Hospitals in the Vermont Oxford Network, with rates of LOS decreasing from 21% in 2000 to 15% by 2009 [5]. A similar analysis of LOS in preterm infants born at <32 weeks gestation in 29 NICUs in the Canadian Neonatal Network showed that 15% of infants developed LOS, with 80% of these infection being gram-positive, chiefly CONS [6].

The incidence of LOS in late preterm infants, born at 34 to 36 weeks gestational age and in term infants is much lower. A large study of more than 100,000 late preterm infants admitted to 248 NICUS in the United States between 1996 and 2007 showed an incidence of 6.3 episodes of LOS per 1000 NICU admissions; with 59.4% caused by gram-positive organisms, predominantly CONS, 30.7% by gram-negative organisms and 7.7% by yeast [7]. In term infants (≥37 weeks gestational age) discharged from NICUs from 1997 to 2010, the rate of late-onset bloodstream infections was 2.7/1000 admissions, with similar pathogens [8].

Apart from prematurity, prolonged duration of parenteral alimentation with delayed enteral nutrition, intravascular catheterization, extended respiratory support on ventilators, gastrointestinal surgery, and use of broad spectrum antibiotics are recognized risk factors for neonatal NI [2]. The very devices that sustain life and provide sustenance to premature and/or sick newborns admitted to the NICU may become channels for bacterial invasion, with fragile skin, and immaturity of immune systems exacerbating the risk.

The most common NI in NICUs are bloodstream infections, often catheter -related (central line associated bloodstream infection, CLABSI), followed by Ventilator-Associated Pneumonias (VAP), surgical site infections and less frequently catheter associated urinary tract infections, and ventricular shunt infections. Skin and soft tissue infections may also be hospital acquired in newborn infants [9].

Outbreaks of NI have been related to overcrowding, understaffing, and contamination of equipment, environment, medications, and even breast milk [10–13].

### Organisms responsible for infections

The microorganisms responsible for NI may be the patient’s own microflora, present on the skin, nasopharynx and gastrointestinal tract, or the transmission of microorganisms from visitors and caretakers. Recent studies have shown that infants with a less diverse gut microbiome harbor pathogenic bacteria in the gastrointestinal tract which may translocate across the epithelial barrier, predisposing them to late-onset bloodstream infections [14, 15].

Table 1 shows the distribution of organisms responsible for LOS in NICHD Neonatal Network NICUs over the years. In resource-limited countries, gram-negative bacteria such as E. Coli, Klebsiella, Acinetobacter and Pseudomonas are the predominant bacteria responsible for NI in neonatal units, and a very high prevalence of antibiotic resistance has been described [16].

Although much attention has been paid to hospital acquired bacterial infections, with the availability of better diagnostic methods, nosocomial viral infections are increasingly being recognized. Respiratory syncytial virus, influenza and parainfluenza viruses are well known for nosocomial transmission, but rhinovirus has recently been identified as an important nosocomial pathogen in preterm infants [17]. Nosocomial viral respiratory infections result in escalation of respiratory support, prolonging length of stay, hospitalization costs and also lead to affected infants requiring home oxygen twice as often as unaffected infants [17]. Rotavirus, adenovirus and norovirus have been responsible for outbreaks of gastrointestinal illness in NICU patients, and have been implicated in clusters of NEC cases [18]. Human parechovirus infections can present with sepsis like syndromes, indistinguishable from bacterial infection, and with symptoms of meningoencephalitis. In a prospective cohort study of preterm infants with suspected LOS over an 18 month period, 13% of infants tested were found to have evidence of parechovirus by reverse transcriptase polymerase chain reaction, confirmed by DNA sequencing [19].

### Prevention of neonatal NI

The “All or None” approach of process quality is an important concept to understand and implement in prevention of NI [20]. NI are usually multifactorial and preventative strategies entail multiple interventions or a series of steps which operate synergistically. Partial execution of a series of steps may be ineffective. For example, insertion of a central line using strict aseptic techniques would be vitiated by improper line care, resulting in CLABSI. Hence, the proposal of “bundles” – a set of evidence based processes, that when instituted as a group, improve outcomes. This has been found to be particularly effective in reducing CLABSIs in NICUs [21].

Among the interventions to prevent neonatal NI, some that appear quite simple (hand hygiene, feeding maternal breast milk) have been shown to be surprisingly effective, while others have not lived up to their theoretical promise (intravenous immunoglobulin), and a few are still being evaluated (lactoferrin). The cornerstone of infection prevention in any setting is hand hygiene.

### Hand hygiene

Hand hygiene is the single most important intervention in interrupting the transmission of microorganisms and thus preventing NI. Bacterial counts on hands of health care workers range from 3.9 × 10^4^ to 4.6 × 10^6^ colony forming units/cm^2^, and may include pathogens such as staphylococcus aureus, klebsiella pneumoniae, enterobacter, acinetobacter and candida [22]. Viable organisms are present on the skin squames that humans shed daily, and these contaminate patient clothing, bed linen and furniture, with transmission by health care workers’ hands if they are not cleaned before and after patient contact. Although this intervention appears simple, implementation is often more challenging than expected, with low compliance rates even in intensive care areas [21]. There is now a global effort to improve hand hygiene compliance with the WHO “Clean Care is Safer Care” campaign [23]. A multipronged effort is required to improve compliance, with education of education of health care workers, performance feedback, reminders, use of automated sinks and introduction of an alcohol based hand rub [24]. It is believed that the introduction of the alcohol based hand rub has revolutionized hand hygiene practice, since it takes less time, improves compliance and has shown to be effective in many settings. Table 2 illustrates the mode of action and efficacy of commodities commonly used for hand hygiene. Apart from health care workers, parents and siblings may also be responsible for transmission of infection [25], so hand hygiene should be emphasized for all visitors/caregivers in the NICU.

**Table 2:** Hand hygiene: materials and efficacy

Agent	Plain soap	Antimicrobial soap with chlorhexidine	Alcohol based hand sanitizer
Mode of action	Detergent effect and mechanical friction	Cationic bisguanide, disrupts cell membranes	Disrupts membranes, denatures proteins, cell lysis
Reduction of bacterial load on hands	0.6 to 1.1 log_10_ CFU	2.1 to 3.0 log_10_ CFU; has persistent residual antiseptic activity on the skin which may last up to 30 min.	3.2 to 5.8 log_10_ CFU
Effective against	Dirt, organic material	Gram-positive cocci	Gram-positive cocci, gram-negative bacilli, mycobacterium tuberculosis, fungi, viruses
Less effective against		Gram-negative bacilli, fungi and viruses, mycobacteria, spore forming bacteria such as Clostridium difficile	Clostridium difficile, Hepatitis A, rotavirus, enteroviruses, adenovirus, spores
Comments	Trauma caused by frequent skin washing may lead to chapping of skin and shedding of resistant flora		Optimal antimicrobial activity at concentration of 60–90%

(from ref [21] and [28])

Artificial finger nails worn by health care providers have been associated with persistent carriage of Pseudomonas aeruginosa, Klebsiella pneumoniae and fungi, and linked to outbreaks with these organisms in intensive care settings [26, 27]. The Hospital Infection Control Practices Advisory Committee (HICPAC) guidelines recommend that health care providers with direct patient contact in intensive care areas should not wear artificial nails [28]. It is unclear if the use of nail polish is associated with NI [29].

### Early feeding and human milk

Since the seminal paper by Narayanan et al. in 1984 that showed that feeding raw unpasteurized maternal milk was associated with lower rates of sepsis in low birth weight infants in India [30], numerous studies in industrialized countries have confirmed that feeding human milk is associated with lower rates of sepsis and necrotizing enterocolitis in preterm and very low birth weight infants [31–33]. Early enteral feeding, within 2 to 3 days of life, has been associated with lower rates of NI, without increasing rates of necrotizing enterocolitis [34]. In addition, human milk is better tolerated than bovine formula, and is associated with establishment of complete enteral nutrition at a faster rate, allowing early discontinuation of central catheters [35]. The advantages of maternal breast milk in preventing NI have not been duplicated by the use of donor milk [31]. Human milk contains secretory antibodies, phagocytes, lactoferrin and prebiotics which improve host defense and gastrointestinal function. A recent review delineates compositional and bioactive differences between mother’s own milk and donor milk which may account for the differences in outcome [36]. It is important to note that human milk can also be associated with outbreaks of infection in NICUs, either due to milk sharing [13] or contamination of equipment such as milk warmers, or collection pumps [12].

### Central line care

Central venous catheters provide stable intravenous access to sick or low birth weight infants who need long term intravenous nutrition or medications, and umbilical arterial catheters are used for blood sampling and continuous blood pressure monitoring. These central lines are ubiquitous, and usually essential in the NICU, but increase the risk of NI by breaching the protective skin barrier and due to the propensity of many microorganisms to form a biofilm [37]. CLABSI are a subset of NI, defined by the Centers for Disease Control and Prevention’s National Healthcare Safety Network (NHSN) as a bloodstream infection in which the initial positive culture occurs at least 2 days after placement of a central line that is in situ or was removed less than 2 days before the positive culture, and the positive blood culture was not attributable to infection at another site [38]. Evidence based care of central lines has resulted in a decrease of CLABSI over the last decade (Table 3). These care “bundles” are not complicated, but require training, commitment, and constant vigilance to maintain compliance. There is still significant heterogeneity in CLABSI prevention practices in NICUs in the United States, and in other countries, with some centers using chlorhexidine for skin antisepsis or for dressings and some centers restricting the use of chlorhexidine to larger infants based on United States Food and Drug Administration guidelines [39, 40]. Nevertheless, from 2007 to 2012, rates of CLABSIs decreased in NICUs in the United States from 4.9 to 1.5 per 1000 central line days [41], with some centers achieving sustained reductions to zero rates [42, 43]. In lower resource countries, CLABSI rates in NICUs participating in the International Nosocomial Infection Control Consortium are reported to be 10 to 20 times higher than those in NICUs reporting data to the CDC NHSN [44].

**Table 3:** Guidelines for prevention of intravascular catheter associated infections

Education and training:	
Educate health care personnel regarding indications for intravascular catheter use, proper procedures for the insertion and maintenance of intravascular catheters and appropriate infection control measures	
Periodically reassess knowledge of and adherence to guidelines for all personnel involved in the insertion and maintenance of intravascular catheters	
Designate only trained personnel who demonstrate competence for the insertion and maintenance of central intravascular catheters.	
Catheter placement and duration of use	
Weigh the risks and benefits of placing a central venous catheter.	
Evaluate daily if catheter is still necessary	
Promptly remove any intravascular catheter that is no longer essential	
Remove and do not replace umbilical artery catheters if any signs of catheter-related bloodstream infection, vascular insufficiency in the lower extremities or thrombosis are present. Optimally umbilical catheters should not be left in place > 5 days.	
Remove and do not replace umbilical venous catheters if any signs of CLABSI or thrombosis are present. Umbilical venous catheters should be removed as soon as possible but can be used up to 14 days if managed aseptically.	
Placing catheters	
Hand hygiene should be performed before and after palpating catheter insertion sites as well as before and after inserting, replacing, or dressing an intravascular catheter.	
Maintain aseptic technique for insertion and care of intravascular catheters.	
Maximum sterile barrier precautions including the use of a cap, mask, sterile gown, sterile gloves and a sterile large drape are necessary for the insertion of a central venous catheter.	
A minimum of a cap, mask, sterile gloves and a small sterile fenestrated drape should be used during peripheral arterial catheter insertion.	
Prepare insertion site with povidone iodine/chlorhexidine containing antiseptic (no recommendation can be made about the safety of chlorhexidine in infants < 2 months)	
Use sterile gauze or sterile, transparent semi-permiable dressing to cover catheter site.	
Do not use topical antibiotic ointment or creams on insertion sites because of potential to promote fungal infections and antimicrobial resistance.	
Do not administer systemic antimicrobial prophylaxis routinely before insertion or during use of an intravascular catheter to prevent catheter colonization or CLABSI.	
Dressing catheters	
Use sterile gloves when changing the dressing	
Replace catheter site dressing if the dressing becomes damp, loose or visibly soiled.	
Catheter care	
Use the minimum number of ports or lumens essential for management of the patient	
Do not submerge the catheter or catheter site in water.	
Minimize contamination risk by scrubbing the access port with an appropriate antiseptic (chlorhexidine, povidone iodine, an iodophor, or 70% alcohol) and accessing the port only with sterile devices.	
Replace tubing used to administer blood, blood products, or fat emulsions (those combined with amino acids and glucose or infused separately) within 24 h of initiating the infusion.	

from ref [38]

### Fluconazole prophylaxis

Candida species colonize the skin and mucous membranes of 60% of critically ill neonates and can rapidly progress to invasive infection, with fungal infections being the 3^rd^ most common cause of NI in neonates [45, 46]. Prematurity, low birth weight, use of cephalosporin antibiotics, exposure to more than 2 antibiotics, exposure to H2 blockers, gastrointestinal surgery, parenteral nutrition use >5 days, use of lipid emulsion for >7 days, lack of enteral feeding and presence of a central catheter have all been associated with increased risk of invasive candidiasis, and in extremely low birth weight infants (< 1000 g), invasive candidiasis has been associated with 73% mortality or neurodevelopmetal impairment [47]. Fungal infection accounted for 9% of cases of LOS in VLBW infants in 1996 [1], but more recent studies indicate that invasive candidiasis has decreased in NICUs in the United States since 1997, probably secondary to the widespread use of fluconazole prophylaxis and decreased use of broad spectrum antibacterial antibiotics [48]. In a study of data from 709,325 infants at 322 NICUS managed by the Pediatrix Medical Group from 1997 to 2010, the annual incidence of invasive candidiasis among infants with a birth weight of 750–999 g decreased from 24.2 to 11.6 episodes per 1000 patients, and from 82.7 to 23.8 episodes per 1000 patients among infants with a birth weight <750 g. Fluconazole prophylaxis increased among all VLBW infants over the years, with the largest increase among infants weighing <750 g at birth, increasing from 3.8 per 1000 infants in 1997 to 110.6 per 1000 infants in 2010. The use of broad spectrum antibacterial antibiotics decreased concomitantly in all infants, from 275.7 per 1000 patients in 1997 to 48.5 per 1000 patients in 2010 [48].

Prophylactic antifungal therapy reduces colonization of the skin, gastrointestinal and respiratory tracts and prevents invasive candida infection in high risk preterm infants [46, 47, 49]. Prophylaxis with intravenous fluconazole at 3 mg/kg twice a week, has been recommended for preterm infants with birth weight < 1000 g or gestational age ≤ 27 weeks gestation, starting within the first 2 days after birth, and continued until there is no necessity for central and peripheral intravenous access. In infants weighing 1000–1500 g, prophylaxis may be considered by individual NICUs with high rates of invasive candidiasis [49]. There has been no evidence of development of resistance to fluconazole with this regimen in neonates, although increasing fluconazole resistance has been documented in adult intensive care units. Oral nystatin has also been shown to be effective for prophylaxis [50], but it cannot be used when infants have ileus, necrotizing enterocolitis or intestinal perforation, all conditions with a high risk of invasive candida infection.

The use of routine fluconazole prophylaxis has been challenged more recently, in a randomized controlled trial in infants weighing <750 g at birth, which showed that although invasive candidiasis occurred less frequently in the fluconazole group (3% [95% CI: 1 to 6%]) versus the placebo group (9% [95% CI: 5–14%]), there was no difference in the composite endpoint of death and invasive candidiasis or in the rates of neurodevelopmental impairment [51].

### Use of topical emollients

Topical emollients such as vegetable oils or aquaphor have been postulated to improve skin integrity and barrier function and thereby prevent invasive infection. A recent Cochrane meta-analysis of 18 primary publications involving 3089 infants did not provide evidence that the use of emollient therapy prevents invasive infection or death in preterm infants in high, middle or low income settings [52].

### Ventilator-Associated Pneumonia (VAP)

Ventilator-associated pneumonia is defined by using a combination of clinical, radiologic and laboratory criteria in a patient who has been on assisted ventilation through an endotracheal or tracheostomy tube for at least 48 h before the onset of illness. However, these criteria are subjective and frequently have common characteristics with other diseases, particularly in low birth weight infants with chronic lung disease. Rates of VAP range from 0 to more than 50 per 1000 ventilator days in various publications, reflecting differences in study patients and definitions. It is also unclear if cultures of tracheal secretions are truly representative of VAP or only indicate colonization. In 2012, the Neonatal and Pediatric Ventilator Associated Events working group recognized that the current VAP surveillance definition is of questionable utility and meaning in the neonatal population and refinements are being sought [53]. While absolute definitions may be lacking, it is well known that endotracheal intubation leads to impairment of mucociliary clearance and the potential for colonization of the endotracheal tube and trachea, from both endogenous and exogenous sources, which may then descend further and result in pneumonitis [54]. Endogenous sources of colonization are oropharyngeal secretions, and aspiration of stomach contents. Exogenous sources include transmission of infection from a health care workers’ hands, contamination of suction apparatus, airway circuits, humidifiers, etc. In neonatal patients diagnosed with VAP, polymicrobial and gram-negative organisms appear to be predominant, although staphylococcus aureus and candida have also been noted [55].

VAP prevention “bundles”, similar to CLABSI prevention bundles, have been used in adult ICUs with success, but many of the interventions are not applicable in neonates [54]. Interventions with potential benefit in neonates are indicated in Table 4. There are few studies showing the impact of infection control measures in reducing VAP rates in NICUs [56, 57].

**Table 4:** Interventions to prevent VAP in Neonates

Definite or probable benefit	Unclear benefit
Caregiver education	Oral care with antiseptic or colostrum
Hand hygiene	Elevation of head of bed
Wear gloves when in contact with secretions	In-Line (closed) suctioning
Minimize days of ventilation	
Prevent unplanned extubation-avoid reintubation	
Suction orophaynx	
Prevent gastric distension	
Change ventilator circuit only when visibly soiled or malfunctioning	
Remove condensate from ventilator circuit frequently	

Modified from ref [54]

### Adjuvant therapy

#### Immunoglobulin therapy

Preterm infants are deficient in immunoglobulin G (IgG) since transplacental transport of maternal IgG is truncated by early delivery, and endogenous production starts only around the third month of life. Polyclonal intravenous immunoglobulin (IVIG) has been evaluated to determine if passive immunotherapy is efficacious in preventing neonatal NI in preterm or low birth weight (<2500 g birth weight) patients. A 2013 Cochrane review summarizing 19 studies enrolling almost 5000 preterm and/or low birth weight patients concluded that when all studies were combined, there was a 3% reduction in sepsis and a 4% reduction in one or more episodes of any serious infection, but was not associated with reductions in other clinically important outcomes, including mortality [58]. The Cochrane review’s final statement was “the decision to use prophylactic IVIG will depend on the costs and the values assigned to the clinical outcomes”, and that no additional trials to test the efficacy of previously studied IVIG preparations are warranted.

Since Staphylococci, especially CONS, are responsible for the majority of late-onset infections in VLBW infants, IVIG preparations containing various type specific antibodies targeting different antigenic sites were developed, but studies of these products (Veronate or INH-A21: antibody against microbial surface components recognizing adhesive matrix molecules, Altastaph: antibody against capsular polysaccharide antigen type 5 and 8, and Pagibaximab: anti-lipoteichoic human chimeric monoclonal antibody) have also shown disappointing results [59, 60]. IgM-enriched immunoglobulins are being evaluated as adjuvant therapy for VLBW infants with proven sepsis, but not for prophylaxis [61].

### Lactoferrin

Lactoferrin is an iron – binding glycoprotein present in mature human milk at a concentration of 1 to 3 g/L and in colostrum at 7 g/L. Lactoferrin limits the amount of iron available to pathogenic bacteria, promotes growth of commensal bacteria, and with lysozyme, another antibacterial present in human milk, is involved in the destruction of gram negative bacteria [36]. Delay in establishing enteral nutrition exacerbates the low lactoferrin levels in preterm infants. Bovine lactoferrin, which is 70% homologous with human lactoferrin, has high antimicrobial activity, and is available commercially as a food supplement, has shown promise in reducing the incidence of late-onset sepsis in VLBW infants, particularly in infants weighing <1000 g at birth [62]. There are ongoing trials which may provide additional evidence of the effectiveness of this intervention before this becomes common practice [63].

### Probiotics

In babies born at term by vaginal delivery, the gut is colonized with probiotic bacteria from the mother such as lactobacilli and bifidobacteria which are crucial to the development of the intestinal mucosal immune system. Preterm neonates have abnormal intestinal colonization, often with pathogenic bacteria and have low numbers of probiotic bacteria. Efforts to repopulate the preterm infant’s gut with probiotics in an effort to decrease late-onset sepsis have resulted in variable success, and metanalysis of trials have given inconsistent results. A Cochrane metanalysis in 2014 of 16 eligible trials with 5338 patients concluded that probiotic supplementation did not result in statistically significant reduction of LOS in preterm infants [64]. A more recent metanalysis of 37 randomized controlled trials with 9416 patients showed that probiotics significantly reduced the risk of LOS (13.9% versus 16.3%, number needed to treat =44), but of all the studies analyzed, the two largest trials did not show a significant reduction in the rates of LOS with probiotics [65]. In the ProPrems study [66], 1099 preterm VLBW infants in Australia and New Zealand were randomized to receive a probiotic combination of Bifidobacterium infantis, Streptococcus thermophilus and Bifidobacterium lactis or placebo. Breast milk feeding rates were high (96.9%) among these infants. No significant difference was found in definite late onset sepsis or all cause mortality, but the rate of Stage 2 necrotizing enterocolitis was reduced (2% versus 4.4%). The Probiotics in Preterm Infants Study Collaborative (PiPs trial) [67] in the United Kingdom recruited 1315 infants of whom 650 were administered the probiotic Bifidobacteium breve BBG-001. There was no significant difference in the incidence of LOS in the probiotic patients (11%) versus the controls (12%) and the rates of NEC were also similar.No adverse effects have been noted in these trials, but there have been case reports of bacteremia in preterm infants originating from probiotic therapy [68]. Despite numerous trials and metanalyses, questions remain about the effectiveness of probiotics, the strains to be used, appropriate dosage, etc.

### Prebiotics

Human milk oligosaccharides (HMO) are complex carbohydrates which promote the growth of beneficial commensals like Bifidobacterium and Bacteroides in the healthy breast fed term infants’ intestine. Most pathogenic Enterobacteriaceae lack specific glucosidases to utilize these oligosaccharides as a food source. In addition, HMOs have structural homology to many cell surface glycans and act as decoys by binding luminal bacteria that are then unable to bind to the luminal enterocytes. HMOs produced by mothers may vary in structure and may influence the intestinal microbiota of their infants [69]. Prebiotics are non-digestible dietary products that selectively stimulate the growth or activity of beneficial commensal bacteria similar to HMOs, but the complexity of this approach to altering gut microbiota is only just beginning to be understood [70]. Synthetic prebiotics such as short chain galacto oligosaccharides, long chain fructo-oligosaccharides, inulin, lactulose are available and have been used in combinations to mimic natural human milk oligosaccharides. A metanalysis of 7 trials including 417 patients showed that supplementation with prebiotics resulted in significantly higher growth of beneficial microbes but did not decrease the incidence of sepsis, NEC or reduce the time to full feeding [71].

### Synbiotics

A synbiotic is a product that contains both a probiotic microbe and a prebiotic substrate. There is experimental evidence that the simultaneous administration of probiotics and prebiotics can improve survival of the probiotic bacteria, but there is no clinical evidence yet that synbiotics are useful in preventing neonatal NI [72].

### Antibiotic stewardship

Empirical antibiotic use is widespread in neonatal intensive care units. A recent review of over 50,000 patients in 127 California NICUs showed a 40 fold variation in antibiotic prescribing practices, despite similar burdens of proven infections, NEC, surgical volume and mortality [73]. Prolonged initial empirical antibiotic treatment in preterm infants has been associated with increased rates of LOS, NEC and death, with each additional empirical treatment day associated with measureable increase in risk [74, 75]. Perinatal and early empiric antibiotic use has been associated with lower bacterial diversity in the developing microbiome of the neonate, and increased colonization with potentially pathogenic Enterobacteriaceae, which may precede bloodstream infection in preterm infants [14, 15, 76, 77]. Widespread antibiotic use, particularly with broad spectrum cephalosporins potentiates the development of resistant strains, and increased colonization and invasive disease due to candida [78]. The gravity of this scenario has been recognized and given impetus to develop local and national antibiotic stewardship programs [79]. However, a prospective longitudinal study of neonatal infections and antibiotic use over 25 years in a tertiary NICU showed that emergence of cephalosporin resistant gram-negative bacterial infection was not prevented by responsible antibiotic use, indicating that the relationship between antimicrobial use and drug resistance is complex and that other factors may be involved [80].

### Management of neonatal nosocomial infections

Infants in the NICU may deteriorate rapidly when they develop NI, so vigilance and high index of suspicion for sepsis is essential. Management includes appropriate diagnostic tests including blood, and whenever possible, cerebrospinal fluid cultures, followed by antibiotic therapy and supportive care.

Initial antibiotic therapy is empirical and targeted against the most likely organisms, based on the clinical presentation, available epidemiological information on the pathogen profile in the neonatal unit where the patient is being treated and in the community [81, 82]. Antibiotic therapy should be narrowed down or modified as soon as culture and antibiotic susceptibility results are available. In infants suspected to have CLABSI, most NICUs use a regimen of vancomycin and gentamicin as initial therapy, to cover the possibility of CONS or a gram-negative infection. However, the use and overuse of vancomycin as the first line of treatment for suspected LOS in NICU patients has led to the emergence of vancomycin resistant enterococci. There is a recommendation that neonatal units consider starting empirical treatment with oxacillin or flucloxacillin instead of vancomycin, together with an aminoglycoside such as gentamicin in infants who are suspected to have CLABSI, but are not severely ill, since CONS sepsis is rarely severe and there would be time to switch to vancomycin if the strain is resistant to the initial treatment [82]. There is no evidence that a delay in vancomycin therapy increases mortality in infants with CONS sepsis [83]. On the other hand, inadequate empirical therapy for MRSA bloodstream infection has been associated with increased mortality, so the judicious selection of initial antibiotics remains critical, but still challenging, since clinical signs are usually non-specific [84]. Gram-negative septicemia and candidemia are often associated with hypotension, thrombocytopenia and acidosis. When gram-negative sepsis is strongly suspected or confirmed, or in the presence of gram-negative meningitis, the addition or substitution of a 3^rd^ generation cephalosporin is justified. Pipercillin–tazobactam may be considered to provide coverage for resistant gram-negative organisms. In infections with extended spectrum beta-lactamase (ESBL) producing organisms, or in critically ill infants with complicated intra-abdominal infections, a carbapenem antibiotic may be considered [82, 85]. A combination of antibiotics is usually used in critically ill infants with necrotizing enterocolitis (NEC) or complicated intra-abdominal infections, where polymicrobial infection with aerobic and anaerobic microorganisms is probable [85]. Results of the ongoing Phase 2/3 study (SCAMP study, NCT 01994993) of different antibiotic regimens for complicated intra-abdominal infections in infants may help guide future therapy. Anaerobic therapy with clindamycin, metronidazole, carbapenems etc. in infants with NEC has been associated with an increase in intestinal strictures, but with lower mortality in infants with surgical NEC [86]. Invasive neonatal candidiasis is treated with amphotericin B deoxycholate, fluconazole or micafungin [87], although some authors suggest reserving fluconazole only for prophylaxis and using amphotericin for treatment to prevent the emergence of resistant strains [49].

In addition to appropriate antibiotics, consideration should be given to removal of central lines since there is an increased risk of infectious complications and persistently positive cultures if the central line is not removed promptly in bacteremic patients or in patients with candidemia [88]. One positive blood culture for Staph aureus, or Gram-negative rods or Candida warrants removal of the central line. Medical management without central line removal may be considered if there is one positive CONS culture, but if cultures are repeatedly positive, the central catheter should be removed, with placement of a new catheter if required, once cultures are negative.

Supportive care for infants with NI includes respiratory, hemodynamic, hematological and nutritional support in the NICU, and close follow up post–discharge with early intervention services since these infants are at increased risk for neurodevelopmental delays [89].

### Control of outbreaks

Apart from endemic infections, outbreaks of bacterial, fungal and viral infections have been reported in NICUs, with serious consequences for patients, huge economic burdens and staffing issues [10, 11, 90]. An analysis of a world wide database of health care associated infections (https://www.outbreak-database.com) showed that NICUs account for 38% of outbreaks in ICUs and 18% of all published outbreaks, and this probably represents only the tip of the iceberg [10]. Klebsiella, Staphylococcus, including MRSA, Serratia, and Enterobacter species were responsible for the majority of reported outbreaks. ESBL producing Enterobacteriaceae have emerged as major pathogens responsible for outbreaks of infection in NICUs with associated significant mortality [11]. A recent review of 75 studies reported 1185 cases of colonization and 860 infected with 16% mortality in infected infants. Klebsiella pneumoniae was the most frequently implicated pathogen. The source of the outbreak was unknown in 57% of the reports; the most commonly identified source was admission of an ESBL colonized infant with subsequent horizontal dissemination. Understaffing was identified as a major risk factor in most studies, but the intervention most commonly implemented to terminate outbreaks was enhanced infection control measures including hand hygiene, contact precautions, patient cohorting/isolation, and environmental cleaning. In 23% of reports, the outbreak of ESBL infection led to ward closure. Most units do not routinely screen infants for ESBL infection. Some countries have adopted more rigorous routine screening measures to identify infants colonized with pathogens, in order to prevent horizontal transmission [91].

Staphylococcus aureus is the second most common cause of late-onset sepsis in VLBW infants [3], and is often implicated in outbreaks [10]. Neonates quickly become colonized after birth from their adult caregivers, and colonization may be precursor to invasive infection. In one study, 34% of mothers were colonized with Staphylococcus aureus, and, the cumulative incidence of S. aureus acquisition in infants born to carrier mothers was 42.6/100 within 72–100 h after birth, rising to 69.7/100 at 1 month follow up [92]. The emergence and rapid rise of methicillin resistant staphylococcus aureus (MRSA) infections caused considerable alarm, but large studies have shown that methicillin sensitive staphylococcus aureus (MSSA) causes more infections and more deaths than MRSA and infection prevention strategies should consider MSSA as well as MRSA [93]. Strategies to prevent MRSA transmission in NICUs have included identifying and cohorting colonized neonates, placing them on contact precautions, enhanced hand hygiene compliance, decolonization of colonized neonates and/or health care workers with topical mupirocin, and use of chlorhexidine baths for patients as well as for health care workers [94]. Following two outbreaks of Staphyloccus aureus infections, one NICU instituted a regimen of prophylactic mupirocin applied to all infants admitted to the NICU throughout hospitalization and found that both MRSA and MSSA colonization decreased from 60% to < 5% [95]. In another level 3 NICU, rigorous attempts at preventing colonization and transmission were inadequate with infants developing infection before being identified as colonized or after attempting decolonization [96].

## Conclusion

The prevention and treatment of nosocomial infections continues to be a complex process with no easy solutions. There have been improvements in some areas with documented improvement in rates of CLABSI, but a number of infections remain difficult to control or eradicate. There are isolated case reports as well as outbreaks of infection with increasingly resistant strains or infections with unusual pathogens. Although there are limitations to the diagnostic and therapeutic arsenal available presently to tackle these infections, much can be achieved by attention to simple preventive measures such as hand hygiene and use of maternal breast milk.

## Abbreviations

CLABSI: Central line associated bloodstream infection
CONS: Coagulase-negative staphylococcus
ESBL: Extended spectrum beta-lactamase
HMO: Human milk oligosaccharides
LOS: Late-onset sepsis
MDRO: Multi-drug resistant organisms
MRSA: Methicillin resistant staphylococcus aureus
MSSA: Methicillin sensitive staphylococcus aureus
NEC: Necrotizing enterocolitis
NI: Nosocomial infections
NICHD NRN: National Institute of Child Health and Human Development Neonatal Research Network
NICU: Neonatal intensive care unit
VAP: Ventilator-associated pneumonia
VLBW: Very low birth weight (birth weight <1500 g)
